# Increased dosage of infliximab is a potential cause of *Pneumocystis carinii* pneumonia

**DOI:** 10.1186/s13099-016-0086-4

**Published:** 2016-02-02

**Authors:** Takuya Iwama, Aki Sakatani, Mikihiro Fujiya, Kazuyuki Tanaka, Shugo Fujibayashi, Yoshiki Nomura, Nobuhiro Ueno, Shin Kashima, Takuma Gotoh, Junpei Sasajima, Kentaro Moriichi, Katsuya Ikuta

**Affiliations:** Department of Medicine, Division of Gastroenterology and Hematology/Oncology, Asahikawa Medical University, 2-1 Midorigaoka-higashi, Asahikawa, Hokkaido 078-8510 Japan

## Abstract

**Methods:**

*Pneumocystis carinii* pneumonia occasionally appears in immunodeficient patients. While several reports have shown that *Pneumocystis carinii* pneumonia occurred in the early phase of starting infliximab treatment in patients with Crohn’s disease (CD), the present case suggests for the first time that an increased dosage of infliximab may also lead to pneumonia.

**Results:**

A 51-year-old male had been taking 5 mg of infliximab for the treatment of CD for 10 years with no adverse events. Beginning in September 2013, the dose of infliximab had to be increased to 10 mg/kg because his status worsened. Thereafter, he complained of a fever and cough, and a CT scan revealed ground-glass opacities in the lower lobes of the bilateral lung with a crazy-paving pattern. Bronchoscopy detected swelling of the tracheal mucosa with obvious dilations of the vessels. A polymerase chain reaction using a bronchoalveolar lavage fluid sample detected specific sequences for *Pneumocystis jirovecii*; thus he was diagnosed with *Pneumocystis carinii* (jirovecii) pneumonia. After discontinuing infliximab and starting antibiotic treatment, his symptoms and CT findings were dramatically improved.

**Conclusions:**

The administration of an increased dosage of infliximab can cause *Pneumocystis carinii* pneumonia in CD patients.

## Background

*Pneumocystis carinii* pneumonia occasionally appears in immunodeficient patients, particularly with the administration of chemotherapy. While several reports have shown that infliximab treatment [1–5], which is generally used for the treatment of rheumatoid arthritis and Crohn’s disease, can lead to *Pneumocystis carinii* pneumonia [6–12], to the best of our knowledge, no case has demonstrated the development of pneumonia due to an increased dosage of infliximab treatment. The present case suggests that an increased dosage of infliximab is a potential cause of *Pneumocystis carinii* pneumonia.

## Case

**Fig. 1:**
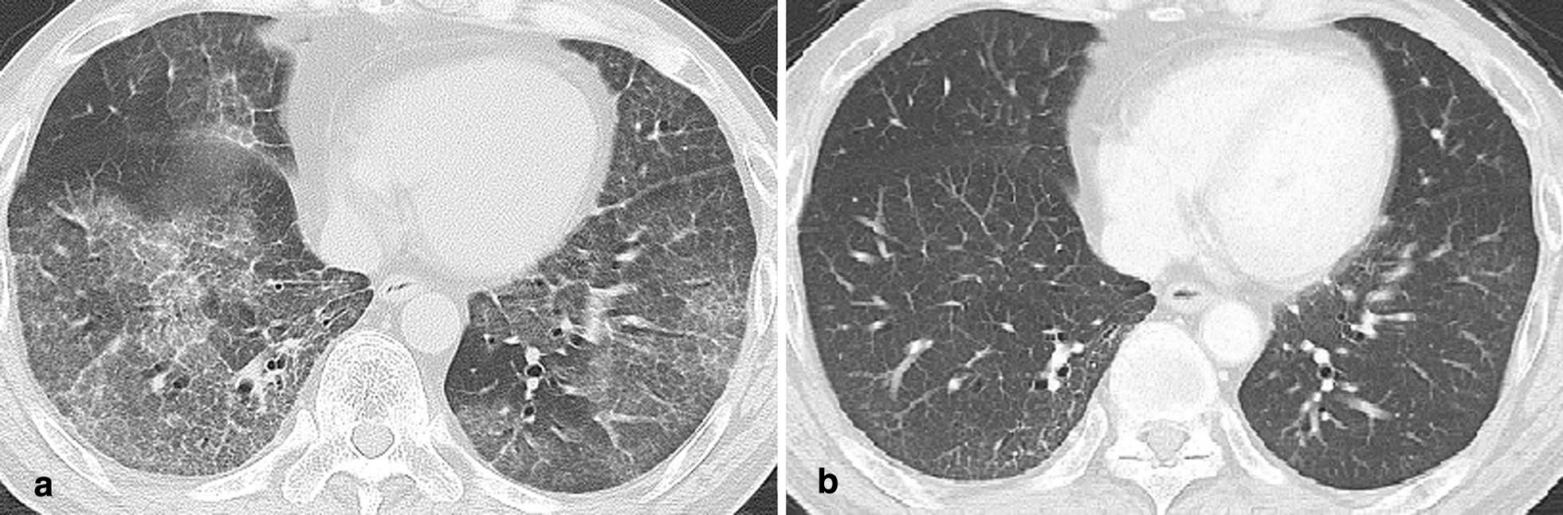
Computed tomography findings of the chest. Ground-glass opacities were seen in the lower lobes of the bilateral lung with a crazy-paving pattern on admission (**a**). Ground-glass opacities were improved after antibiotic therapy (**b**)

**Fig. 2:**
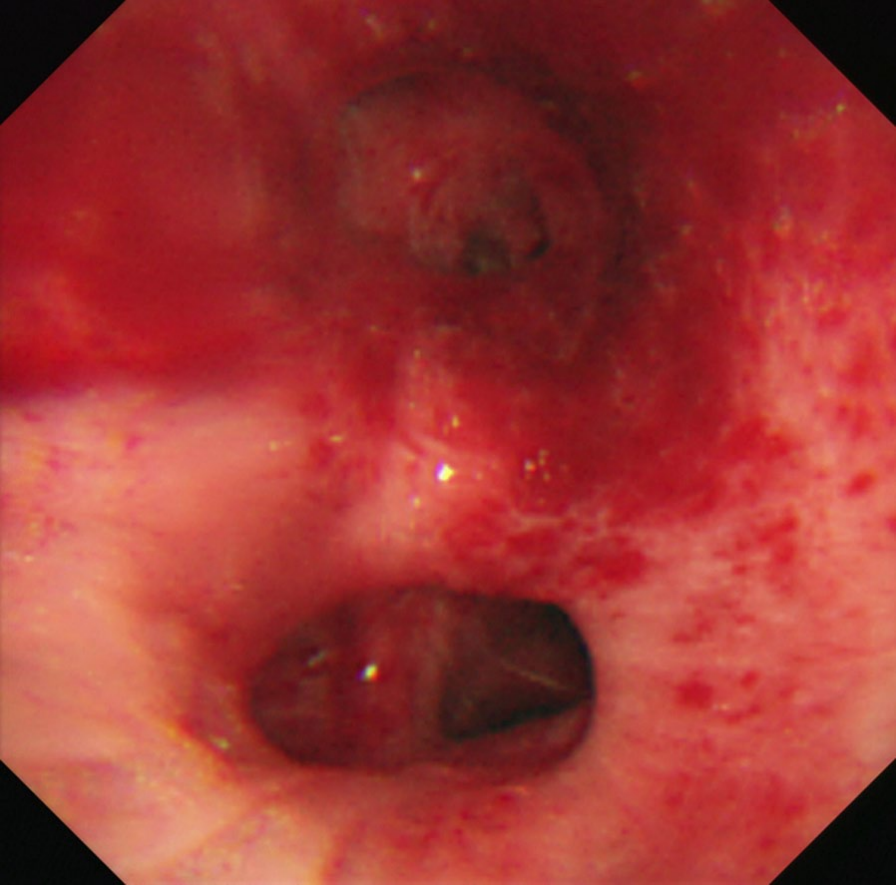
Bronchoscopy finding. Swelling and obvious dilations of the vessels were seen in the tracheal mucosa on admission

A 51-year-old male was suffering Crohn’s disease (CD) since 1990. The patient’s weight was 67 kg. He had the inflammatory type of Crohn’s disease, and the area of his lesion was the colon and small intestine. He had no complications, including intra- or extraenteral lesions, or any other systemic disorders. He had been taking 5 mg/kg of infliximab (total 400 mg) since 2004 and 0.15 g/day of 6-mercaptopurine since 2005. However, he complained of abdominal pain, his Harvey–Bradshaw index scores increased from 5 to 13, and he subsequently began taking 10 mg/kg of infliximab (total 650 mg) from September 2013. In January 2014, he complained of a fever and cough. Laboratory tests on this admission showed high levels of C-reactive protein and anti-mycoplasma antibody while other examination items, including the white blood cell count and T-SPOT, were unremarkable. A computed tomography (CT) scan revealed ground-glass opacities in the lower lobes of the bilateral lung with a crazy-paving pattern (Fig. 1a). Bronchoscopy detected swelling of the tracheal mucosa with obvious dilations of the vessels (Fig. 2). A bronchoalveolar lavage fluid sample obtained during bronchoscopy included 255 cells/field with 80 % macrophages, 12 % lymphocytes and 8 % neutrophils. A polymerase chain reaction using the bronchoalveolar lavage fluid sample detected specific sequences for *Pneumocystis jirovecii*. Taken together, he was diagnosed as having pneumonia due to the combined infection of *Mycoplasma* and *Pneumocystis carinii* (jirovecii). The patient stopped infliximab administration and took 2 g/day of ceftriaxone, 500 mg/day of azithromycin and 400 mg/80 mg/day of sulfamethoxazole/trimethoprim; thereafter his symptoms including a fever and cough improved. CT after the antibiotic therapy showed a significant improvement of the ground-glass opacities in the lower lobes of the bilateral lung (Fig. 1b).

## Discussion

The present report demonstrated a case of *Pneumocystis carinii* pneumonia due to the increased dosage of infliximab. While several cases have demonstrated pneumonia due to *Pneumocystis carinii* after infliximab treatment, to the best of our knowledge, this is the first case in which an increased dosage of infliximab triggered pneumonia. A summary of previously reported cases and our case of *Pneumocystis carinii* pneumonia in CD patients is shown in Table 1.

**Table 1:** Date on pneumocystis pneumonia during administrarion of infliximab

No.	Reference	Year	Sex	Age at diagnosis (years)	Crohn’s disease duration	IFX duration	Dose of IFX	Concomitant drug(s)	Medication	Clinical course
1	Seddik et al. [6]	2004	Male	29	ND	1 month	5 mg/kg	PSL + AZA	ST	2 weeks alive
2	Velayos et al. [7]	2004	Male	19	2 years	2 years 3 months	5 mg/kg	AZA	ST + PSL	2 weeks alive
3	Kaur et al. [8]	2004	Male	59	3 weeks	9 weeks	ND	PSL	ST + PM	1 month died
4	Stratakos et al. [9]	2005	Female	77	9 months	8 months	5 mg/kg	mPSL + AZA	ST	6 month alive
5	Itaba et al. [10]	2007	Female	57	21 years	5 weeks	5 mg/kg	PSL + AZA	ST + mPSL	4 month alive
6	金井 et al. [11]	2009	Male	69	44 years	4 weeks	5 mg/kg	5-ASA + PSL	ST + mPSL	6 month alive
7	Tshudy et al. [12]	2010	Male	8	6 years	15 months	5 mg/kg	None	ST	4 weeks alive
8	Present case	2014	Male	51	24 years	10 years	10 mg/kg	6-MP (0.15 g/day)	ST	1 year 5 month alive

*ST* sulfamethoxazole/trimethoprim, *PSL* prednisolone, *mPSL* methylprednisolone, *PM* pentamidine, *AZA* azathioprine, *5*-*ASA* mesalazine, *6*-*MP* 6mercaptopurine, *ND* not described

Of the seven cases, five were male and two were female. The age ranged from 8 to 77 years. While *Pneumocystis carinii* pneumonia appeared at 4 weeks to 29 months after starting infliximab treatment in previous reports, the present case exhibited pneumonia at 120 months after starting infliximab treatment, at 102 months after starting the 6-mercaptopurine treatment, 24 weeks after starting an increased dosage of infliximab from 5 to 10 mg/kg. This suggests that an increased dosage of infliximab is a potential cause of immunodeficiency, leading to *Pneumocystis carinii* pneumonia. Notably, all cases developing *Pneumocystis carinii* pneumonia took immunomodulators and/or steroids, suggesting that the combined use of these drugs is a risk for pneumonia. Because sulfamethoxazole/trimethoprim was effective for all cases, antibiotics should be immediately administered after the diagnosis of *Pneumocystis carinii* pneumonia.

## Conclusions

The findings of the present case suggest that the administration of an increased dosage of infliximab, as well as a general dose of infliximab, can cause *Pneumocystis carinii* pneumonia in CD patients, particularly in patients taking immunomodulators and/or steroids, illustrating the need for follow up, including pulmonary symptoms and CT examinations, when increasing the dosage of infliximab in CD patients.

## Consent

Written informed consent was obtained from the patient for publication of this case report and accompanying images.
